# Two-Year Variations of Phenolics, Flavonoids and Antioxidant Contents in Acacia Honey

**DOI:** 10.3390/molecules181214694

**Published:** 2013-11-27

**Authors:** Mohammed Moniruzzaman, Siti Amrah Sulaiman, Siti Amirah Mohd Azlan, Siew Hua Gan

**Affiliations:** 1 Department of Pharmacology, School of Medical Sciences, Universiti Sains Malaysia, Kubang Kerian 16150, Kelantan, Malaysia; E-Mails: rasmo04@yahoo.com (M.M.); amylan89@gmail.com (S.A.M.A.); 2 Human Genome Centre, School of Medical Sciences, Universiti Sains Malaysia, Kubang Kerian 16150, Kelantan, Malaysia; E-Mail: shgan@usm.my

**Keywords:** antioxidant activity, phenolics, flavonoids, yearly variations, honey

## Abstract

Honey is a good source of several important chemical compounds and antioxidants and is harvested throughout the year. However, no study has determined how their contents change over the years. The aim of the present research was to investigate the changes in the phenolics, flavonoids and antioxidant properties, as well as other physicochemical properties, of Malaysian acacia honey collected during different months during a two year period. The DPPH (1,1-diphenyl-2-picrylhydrazyl) and FRAP (ferric reducing antioxidant power) methods were used to determine the total antioxidant activity of the honey samples. Generally, honey samples collected in the beginning and the middle of the year tended to have higher sugar content, which may be attributed to its high acidic nature and low moisture content. There was a gradual increase in the phenolic content of the acacia honey samples collected between September 2010 and December 2010. The honey sample collected at the beginning of the year (January) showed the highest color intensity and was dark amber in color. It also contained the highest concentration of phenolic compounds (341.67 ± 2.94 mg_gallic acid_/kg), the highest flavonoid content (113.06 ± 6.18 mg_catechin_/kg) andthe highest percentage of DPPH inhibition and the highest FRAP value, confirming its high antioxidant potential. There was a positive correlation between DPPH and total phenolic content, suggesting that phenolic compounds are the strongest contributing factor to the radical scavenging activity of Malaysian acacia honeys. Overall, our results indicated that there were significant seasonal variations in the antioxidant potentials of honey over the two year period and the time of honey collection affects its physicochemical properties. Therefore, acacia honey from Malaysia should ideally be collected during the dry season, particularly in the months of January, May and June.

## 1. Introduction

Honey is a sweet and flavorful natural product that is valued for its high nutritive value and its contribution to human health for many decades [1]. Approximately 200 substances have been reported to be present in honey, which is considered an important part of traditional medicine [2]. Among the different compounds, a number of components are known to act as antioxidants, including vitamin C, vitamin E, phenolic compounds and enzymes, such as catalase and peroxidase [3]. Honey also contains a variety of phenolic compounds, which are good sources of antioxidants, thereby making honey a good additive and increasing its potential and use in ethnomedicine [3,4,5].

Acacia honey is a monofloral honey produced by *Apis mellifera*, a cultured bee that harvests the extrafloral nectar from the forest mangrove or mangium tree (*Acacia mangium*) [6]. *Acacia mangium* is a species of flowering tree in the pea family of *Fabaceae*. It is widely known as forest mangrove and usually grows up to 30 metres. Acacia honey is one of the most popular honeys in Malaysia and it is usually harvested by the beekeepers and widely consumed all year round. However, its medicinal properties are still under debate; it has previously been reported that honeys that are light in color, including acacia and lime honeys, show lower antioxidant activities when compared to darker honeys, such as forest, chestnut, spruce or fir honeys [7,8]. Due to the diverse and complex composition of honey, samples originating from similar botanical origins may still have different antioxidant contents.

Many authors have demonstrated that honey serves as a source of natural antioxidants and therefore, it is effective in reducing the risk of heart disease, cancer, immune system decline, cataracts and different inflammatory processes [7,9]. In recent years, there has been an increasing interest in determining the antioxidant activity of honey. Many studies have indicated that the antioxidant activity of honey widely varies, depending on the floral source [10]. The botanical origin of honey is reported to have the greatest influence on its antioxidant activity, while the processing, handling and storage affect the honey’s antioxidant activity only to a certain extent [4,5,9].

According to the Malaysian Meteorological Department, Malaysia experiences some seasonality. Generally, the end of the year (November and December) and the beginning of the year (January) receive the most rainfall, especially on the east coast of peninsular Malaysia, such as Kelantan, Terengganu and Pahang. On the other hand, June and July are generally the driest months. February also tends to be dry due to the intermonsoon months.

In a previous study, the antioxidant properties of Brazilian propolis were found to show seasonal variations when investigated over the course of one year [11]. However, to date, the effects of seasonal variations on the antioxidant activity of honeys over the years, as well as the changes in the level of antioxidants, have not been reported. If significant variations in the antioxidant potential of honey exist, then honey should ideally be selectively harvested only during certain months to maximize its antioxidant potential. Thus, the aim of the present study was to evaluate the variations of the antioxidant properties, as well as other physicochemical properties of acacia honey collected at different times over the course of two years.

## 2. Results and Discussion

### 2.1. pH

**Figure 1:**
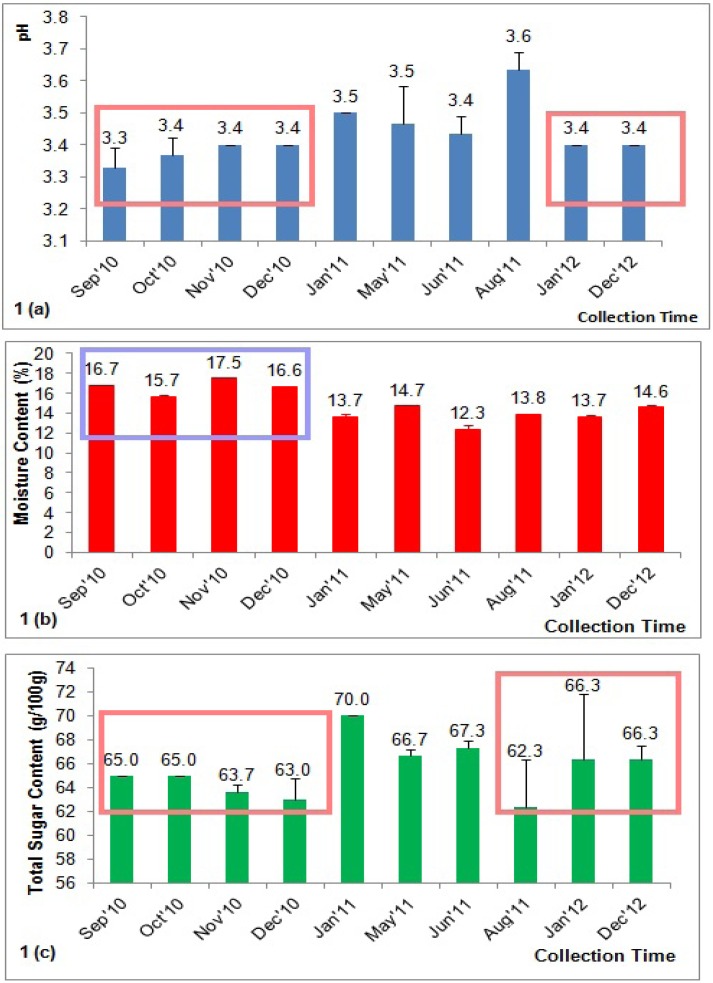
Variations in the (**a**) honey pH, (**b**) moisture content and (**c**) total sugar content in the investigated honey samples.

Overall, the pH values of the honey samples were acidic in nature (pH 3.33–3.63), which were within the recommended limits (pH 3.4 to 6.1) for fresh honey (Figure 1a). Our result was similar to previous reports on the pH of Malaysian acacia honeys (pH 3.40 according to [12] and pH 3.53 according to [6] and also to that of honey samples from Nigeria (pH 3.56) [13]. However, some other honey samples are slightly more alkaline, such as honey from Romania (pH 4.0) [14], Lithuania(pH 3.86) [15], Poland (pH 3.53–4.88) [16] and Germany (pH 5.40) [17]; other honeys are more acidic, such as honey from Pakistan (pH 3.2) [18].

The acidity of the honey is reported to be responsible for two important characteristics of the honey: the flavor and its stability against microbial spoilage [19]. There were significant pH variations among the honeys collected in the different months (Figure 1a). Our results indicated that honey that was collected towards the end of the year during the rainy season, between September 2010 and December 2010, tended to be more acidic. This could be due to the heavy rains during these months, which encompassed the rainy season in Malaysia, contributing to the higher moisture content in the honey (refer to the section on moisture content). Many types of honey have been reported to generate hydrogen peroxide when diluted due to the activation of the glucose oxidase enzyme, which oxidizes glucose to gluconic acid and hydrogen peroxide [20]. However, we do not discount the possibility that the low pH of the investigated honeys may also be attributed to the conversion of the sugar in the honey into organic acids. The variations in the pH value of the acacia honey in this study and in the acacia honey from different countries may also be attributed to variations in storage time, as well as geographical and botanical sources.

### 2.2. Moisture Content

The amount of water present in the honey can easily be determined by measuring the moisture content, which is another important parameter for honey quality. In the present investigation, the moisture content of the acacia honey was between 12.33% and 17.46%, which is within the limit (≤20%) set by the international regulations and confirmed the good quality of the honey (Figure 1b) [21]. This level is comparable to the level found in a recent study of Malaysian acacia honey (15.16%) [6]. Several studies have also reported the moisture content of acacia honey from different parts of the world, such as from Germany (17%) [17], Nigeria (16.45%) [13] and Pakistan (18.7%) [18].

There were significant changes in the moisture content among the different batches of acacia honey (Figure 1b). The acacia honey collected towards the end of the year (September 2010 to December 2010), which included the rainy seasons, showed correspondingly higher moisture contents. The lower moisture content observed in the rest of the samples may be attributed to the hotter weather and the decreased rainfall, especially from the beginning of the year to the middle of the year in Malaysia. However, a lower moisture content is desirable as it contributes to the honey’s ability to resist fermentation and granulation during storage, thus promoting a longer shelf life [21]. This fact may also contribute to the higher acidity that was described for some of the samples (see above).

### 2.3. Total Sugar Content

The total sugar content of the acacia honey was between 62.33 and 70.00 g/100 g, which was within the highest limit (≥60%) determined for total sugar content by the European Community directive [22], indicating the honey’s good quality. In a previous study, acacia honey was determined to be the sweetest among the different types of Malaysian honey, with a total sugar content of 68.4 g/100 g [6]. However, the total sugar content of the investigated acacia honey was still lower than the acacia honey from Pakistan (81.4%) [18], indicating that the geographical origin also influences the total sugar content of honey.

Again, there were significant differences in the total sugar content throughout the collection period. The honey samples collected in the beginning of the year and in the middle of the year tended to have higher sugar contents (Figure 1c). The high sugar content of the investigated honey samples may be attributed to its high acidic nature and low moisture content, both of which inhibit the formation of 5-hydroxymethylfurfural from the sugars, especially glucose and fructose.

### 2.4. Color Characteristics

The color of honey is the primary parameter for its classification based on the USDA-approved color standards [23]. Honey color naturally varies, with a wide range of color from light yellow to amber, as well as dark amber and even black in extreme cases; green or red hues may also occur [24]. The mean mm Pfund value for the investigated acacia honey samples was 120.50 ± 23.46 and most of the acacia honeys were dark amber in color. In a previous study, acacia honey was light amber in color [6], which was similar to some of the samples (AH-1, AH-8 and AH-10) in the present study.

**Figure 2:**
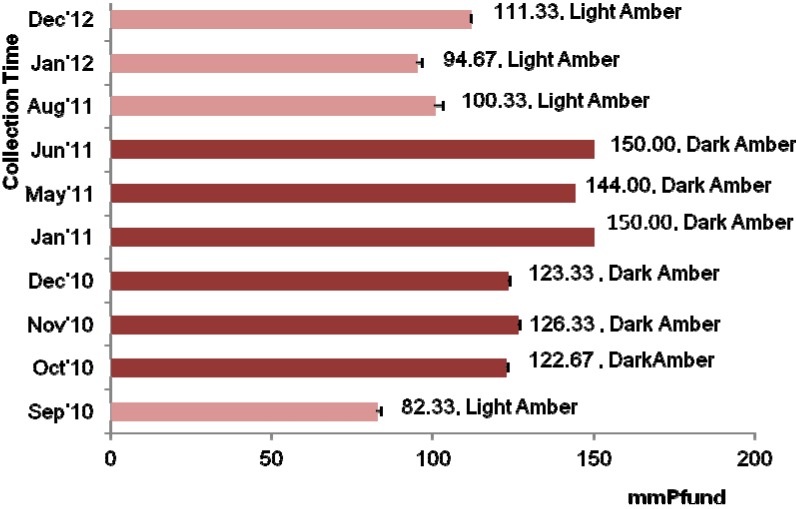
Color characteristics of the Malaysian acacia honeys.

The color of honey samples collected during the middle of the year tended to be dark amber (Figure 2). It is possible that the darker color of the honey collected in the middle of the year was due to the lower moisture content, while the lighter color of the honey was related to its high moisture content, as discussed above. Additionally, it was previously reported that honey darkens with age and other changes in color may result from the beekeeper’s interventions and different conservation methods, such as the use of old honeycombs, contact with metals and exposure to high temperatures or light [23].

### 2.5. Color Intensity

ABS_450_ is an important test to analyze the color intensity of the honey; this measurement is related to the presence of pigments in honey, such as carotenoids, minerals, pollen, phenolics and flavonoids, which are known to exhibit antioxidant properties [25]. The color intensity of the acacia honey in the present study was between 327.67 and 875.67 mAU. The color intensity of these investigated acacia honeys were higher than previously reported acacia honeys from Malaysia (320.33 mAU) [6] and some tualang honeys (297.74 and 489.5 mAU), which are well known in Malaysia for their medicinal properties [26,27]; this higher value indicated the better quality of the investigated acacia honeys in this study.

**Figure 3:**
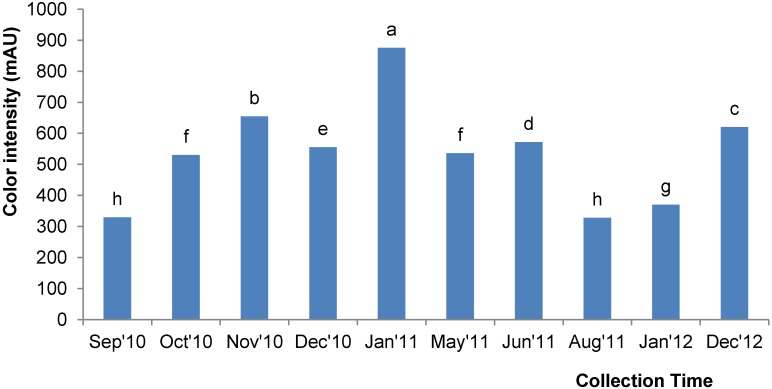
Color intensity of the Malaysian acacia honeys. Values with different letters indicate significant differences (*p <* 0.05).

Again, there was an obvious trend in the color intensity of the honey samples; the samples collected towards the end of the year (between September 2010 and December 2010 as well as those in August 2011 to December 2012) had lower color intensities compared to the honey collected in the beginning (January 2011) or in the middle of the year (Figure 3). The highest color intensity was exhibited by acacia honey sample AH-5, which was collected during January 2011; this high color intensity could be due to its high total phenolic and flavonoid content as described below.

### 2.6. Total Phenolic Content

All of the tested honeys contained high levels of polyphenols, indicating that they were rich in antioxidants. The total phenol content per kg of honey ranged from 129.16 to 341.67 mg_gallic acid_. The mean total phenolic content of the investigated acacia honey sample was 233.84 ± 1.52 mg_gallic acid_/kg of honey. The mean phenolic content in the acacia honey was higher than those obtained in other studies, such as the total phenolic content in acacia honey from Italy (55.2 ± 2.8 mg/kg) [5], Slovenia (25.7 to 67.9 mg/kg) [7], Croatia (31.72 to 80.11 mg/kg) [28], Malaysia (186.70 mg/kg) [6] and Poland (325.40 mg/kg) [29]. However, it was lower than honey from Germany (627.56 ± 44.03 mg/kg) [17] and another sample reported from Poland (405.50 mg/kg) [29]. Generally, the high phenolic content of the investigated Malaysian acacia honey confirmed the good quality of the honey. The variations in the phenolic content of the investigated acacia honey compared to those from different countries may be due to the different geographical and botanical sources of the honey.

**Figure 4:**
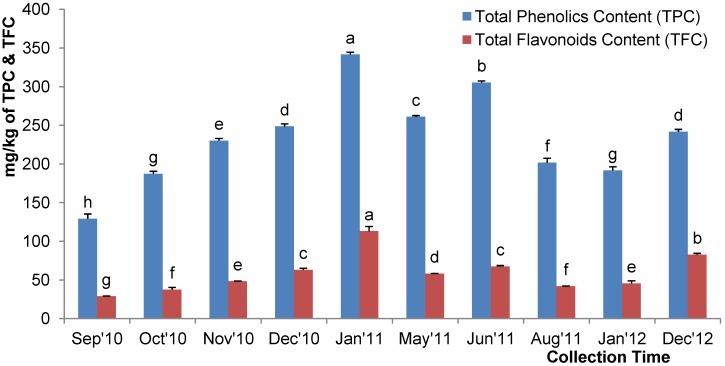
Total phenolic and flavonoid contents of the Malaysian acacia honeys. Values with different letters indicate significant differences (*p <* 0.05).

There were significant differences in the mean total phenol content of the different batches of acacia honey collected during the different months (Figure 4). There was a gradual increase in phenolic content in the acacia honey samples collected between September 2010 and December 2010. The level of phenolic content of the acacia honeys collected in December 2010 (AH-4) was similar to the content of the honey harvested in December 2012 (AH-10), which may be attributed to the similar types of pollen gathered during the same month. Among all of the samples, acacia honey sample AH-5, which exhibited the highest color intensity and was dark amber in color, contained the highest concentration of phenolic compounds (341.67 ± 2.94 mg_gallic acid_/kg). Previously, Blasa *et al.* [8] and Bertoncelj *et al.* [7] reported that darker honeys tended to have higher phenolic contents when compared to lighter colored honey and our findings are in agreement.

### 2.7. Total Flavonoid Content

The total flavonoid content of acacia honey ranged from 28.83–113.06 mg_catechin_/kg of honey (Figure 4). The honey samples that were reported to have high flavonoid content also tended to have correspondingly high phenolic content. This was expected because flavonoids are also derived from phenolic compounds [30]. The flavonoid content of the investigated acacia honey was higher than honey from Romania (0.91 to 2.42 mg/100 g) [31] and Italy (0.45 to 1.01 mg/100 g) [8] but was lower than Burkina Fasan honey (0.17 to 8.35 mg of QE per 100 g of honey) [32].

There were significant seasonal variations among the acacia honey samples investigated in the present study, which indicates the variation in the pollen composition during different times of honey harvesting. The highest flavonoid content (113.06 ± 6.18 mg_catechin_/kg) was found in sample AH-5, which was collected in January 2011; this sample also showed the highest phenolic content and the strongest color intensity. The lowest flavonoid content was observed in the samples collected in September 2010 and the flavonoid levels increased gradually until peaking in January 2011, only to subsequently decrease. Similarly, seasonal variation in the total flavonoid content was also reported in bamboo leaves, where the lowest content was found in May and peaked in January [33].

### 2.8. DPPH Free Radical-Scavenging Activity

DPPH is an unwavering, nitrogen-centered free radical that has been widely employed to test the free radical scavenging ability of various samples, including honeys. In evaluating the radical scavenging potential of honeys, DPPH scavenging activity indicates superior antioxidant activity [5]. The mean DPPH scavenging activity (43.89%) was similar to previously reported Malaysian acacia honeys (42.03%) and Algerian honeys (44.55%) [34]; however, this activity was lower than tualang honeys (59.89%) [6] and Indian honeys (57.5%) [35], all of which may have comparatively higher antioxidant potential when compared to acacia honey.

**Figure 5:**
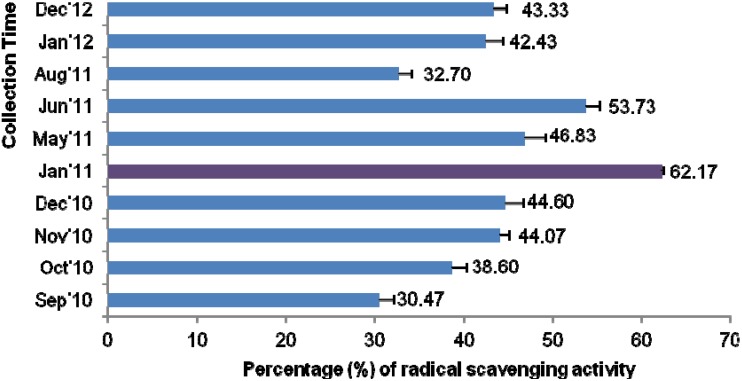
DPPH radical scavenging activities of the Malaysian acacia honeys.

The highest percentage of inhibition was again exhibited by sample AH-5 (62.17%), which was collected in January 2011 (Figure 5). Its high percentage of radical scavenging activity may be due to its higher phenolic and flavonoid contents as the antioxidant potential of honey has been shown to be directly correlated with its phenolic and flavonoid contents [5], where high DPPH scavenging activity showed superior antioxidant activity.

### 2.9. Determination of Total Antioxidant Content by FRAP Assay

**Figure 6:**
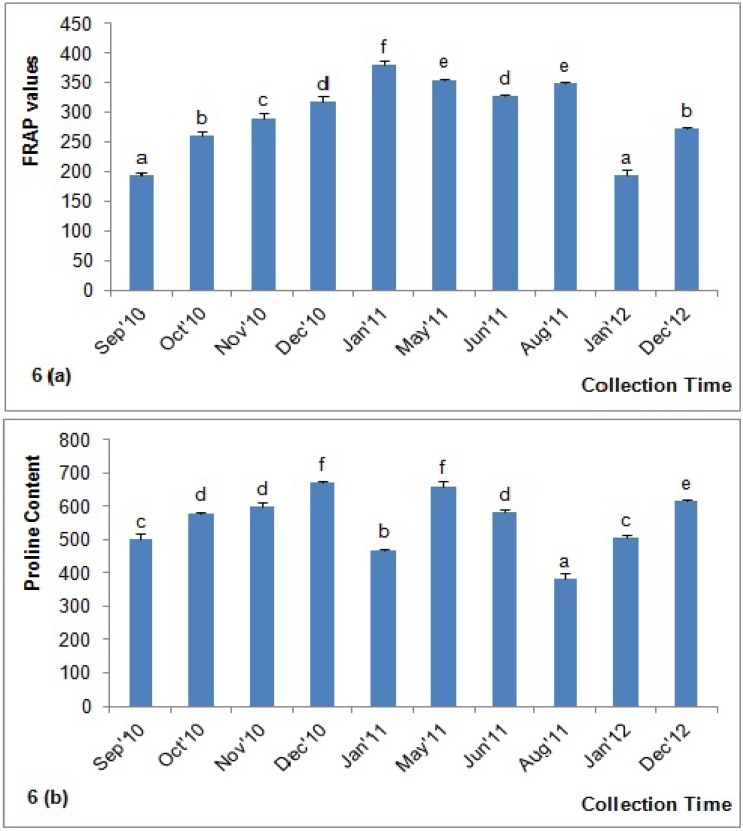
(**a**) FRAP values and (**b**) proline content of the Malaysian Acacia honeys. Values with different letters indicate significant differences (*p <* 0.05).

FRAP is a simple, direct test that is widely used to test antioxidant capacity; this test estimates the amount antioxidants or reductants present in a sample based on their ability to reduce ferric (Fe^3+^) to ferrous (Fe^2+^) compounds [6]. The FRAP values for the investigated acacia honeys were between 193.31 and 379.53 µM Fe (II)/100 g (Figure 6a). Our reported FRAP value for acacia honey was higher than the values previously reported for Malaysian acacia honeys (100.90 ± 2.44 μM Fe (II)/100 g) [6]. Our result was also higher than the FRAP values of acacia honey collected from Croatia, which ranged from 39.53 to 173.46 μM Fe (II) [30] and 72.87 ± 15.44 μM Fe (II) [28], as well as acacia honey from Italy (79.5 ± 3.7 μM Fe(II)) [5].

There were significant differences among the acacia honeys that were collected during the different months (Figure 6a). Sample AH-5, which was collected in the beginning of the year (in January 2011), again showed the highest FRAP value, confirming its high antioxidant potential. The high total phenolic and flavonoid contents may allow this sample to reduce the most Fe^3+^ ions to Fe^2+^ ions, thus yielding the greatest FRAP values.

### 2.10. Proline Content

Proline is an important amino acid that originates mostly from the salivary secretions of *A. mellifera* during the conversion of nectar into honey [36]. Proline content is an indication of honey ripeness and, in some cases, sugar adulteration. According to Bogdanov *et al.* [19], the proline levels in honey should be more than 183 mg/kg, as was found in all of the tested honey samples. The mean proline concentration of the investigated honeys was 555.88 mg/kg. Our result was similar to some Algerian honeys (202 and 680 mg/kg) [37], Indian honeys (133–674 mg/kg) [35] and acacia honeys from Malaysia (517.55 mg/kg) [6]. The higher proline concentration in the investigated honeys confirmed the better quality of these honey types.

There were significant differences in the proline content of the honeys collected during the different months, indicating that seasonal variation may have some effects on the proline content of these honeys (Figure 6b). The highest proline concentration was observed in sample AH-4 (669.87 mg/kg), which was collected in December 2010. High proline concentrations were also observed in honey samples collected in the middle of the year (between May and June 2011), as well as for the sample collected in December 2012.

### 2.11. Correlation

The correlation matrix between the phenolics, flavonoids, DPPH, FRAP and ABS450 indicates a significant correlation between the biochemical and antioxidant parameters (Table 1). A strong correlation was found between the color intensity of the honey samples and the antioxidant parameters, such as flavonoids, phenolics and FRAP values, with r values of 0.842, 0.816 and 0.557, respectively. The strongest correlation existed between the color intensity and both the phenolic (*r* = 0.816) and the flavonoid (*r* = 0.842) contents, indicating that a darker colored honey can be attributed to the presence of high amounts of phenolic and flavonoid compounds, which affect the antioxidant potential of the honey. For instance, acacia honey sample AH-5, which showed the highest color intensity, also contained the highest concentrations of phenolics and flavonoids and this sample most likely had the highest antioxidant potential.

**Table 1:** Correlation matrix showing the correlations between the phenolics, flavonoids, DPPH scavenging, FRAP, and ABS_450_ values.

	Phenolics	Flavonoids	DPPH	FRAP	ABS_450_
**Phenolics**	1.000	0.872 **	0.785 **	0.780 **	0.816 **
**Flavonoids**	0.872 **	1.000	0.840 **	0.595 *	0.842 **
**DPPH**	0.785 **	0.840 **	1.000	0.850 **	0.820 **
**FRAP**	0.780 **	0.595 *	0.850 **	1.000	0.557 *
**ABS_450_**	0.816 **	0.842 **	0.820 **	0.557 *	1.000

* Correlation is significant at the 0.05 level (2-tailed); ** Correlation is significant at the 0.01 level (2-tailed).

In a previous study conducted by Bertoncelj *et al.* [7], a slightly strong correlation between ABS_450_ and FRAP values (*r* = 0.85) was found in Slovenian honeys. In Indian honeys, the correlation between ABS_450_ and FRAP was 0.83 [35]. Thus, the higher correlation in our study indicates that acacia honeys have a stronger antioxidant capacity compared to Indian honeys, but a lower capacity than Slovenian honeys.

A positive significant linear correlation was also observed between the DPPH radical scavenging activity and the phenolic and flavonoid contents, as well as between the FRAP values and the phenolic and flavonoid contents. Overall, the positive correlations between DPPH and total phenolic content suggest that phenolics are the strongest contributing factor to the radical scavenging activity of the investigated Malaysian acacia honeys. Future studies should focus on investigating the various constituents of the honey to determine if they correlate with the observed seasonal changes in higher number of samples. It is also plausible that the variations observed in the analyzed compounds may also be influenced by the flora that surrounds the acacia tree through the different seasons and this needs to be further investigated.

## 3. Experimental

### 3.1. Honey Samples

A total of ten acacia honey (*n* = 10) samples (AH-1 to AH-10) were collected from *Apis mellifera* bees supplied by the MB An-Nur Apiary (J-1-0669), Johor, Malaysia. All honey collections were performed during a two year timespan, between September 2010 and December 2012 (Table 2). The bees collected nectar from the honey dew of wild acacia trees (*Acacia Mangium*) located in the Hulu Sedili Reserved Forest, Kota Tinggi, Johor on the east coast of peninsular Malaysia. The samples were refrigerated (4–5 °C) in airtight plastic containers until further analysis. Each analysis was conducted in triplicate.

**Table 2:** Details of the investigated acacia honey samples.

Type of Honey	Sample ID	Time of Collection
Acacia Batch 1	AH-1	September 2010
Acacia Batch 2	AH-2	October 2010
Acacia Batch 3	AH-3	November 2010
Acacia Batch 4	AH-4	December 2010
Acacia Batch 5	AH-5	January 2011
Acacia Batch 6	AH-6	May 2011
Acacia Batch 7	AH-7	June 2011
Acacia Batch 8	AH-8	August 2011
Acacia Batch 9	AH-9	January 2012
Acacia Batch 10	AH-10	December 2012

### 3.2. Chemicals and Reagents

Catechin, 2,2-diphenyl-1-picrylhydrazyl (DPPH), 2,4,6-tris(1-pyridyl)-1,3,5-triazine (TPTZ), Folin–Ciocalteu’s reagent and gallic acid were purchased from Sigma-Aldrich (St. Louis, MO, USA). Sodium carbonate (Na_2_CO_3_), aluminum chloride (AlCl_3_), sodium nitrite (NaNO_2_) and sodium hydroxide (NaOH) were purchased from Merck (Darmstadt, Germany). All chemicals were of analytical grade.

### 3.3. Physical Analysis

#### 3.3.1. pH

A pH meter (HI 98127, Hanna instruments, Mauritius) was used to measure the pH of a 10% (w/v) solution of honey prepared in Milli-Q water (Millipore Corporation, Billerica, MA, USA).

#### 3.3.2. Moisture Content

The moisture content was determined using a refractometric method; the refractive index tends to increase as the solid content present in the sample increases. The refractive indices of the honey samples were measured at ambient temperature using an Atago handheld refractometer (KRUSS, HRH30, Hamburg, Germany). The measurements were further corrected for the standard temperature of 20 °C by adding a correction factor of 0.00023/°C. The moisture content was measured in triplicate and the percentage of moisture content that corresponded to the corrected refractive index was calculated using Wedmore’s table [38].

#### 3.3.3. Total Sugar Content

The honey was diluted in Milli-Q water to obtain a 25% (w/v) solution. The total sugar content of each honey sample was determined using a refractometric method (Atago handheld refractometer, ATAGO, N-1α, Tokyo, Japan). The percentage of sucrose content is expressed as g/mL of honey.

#### 3.3.4. Honey Color Analysis

The color intensity of the honey samples was measured according to the Pfund classifier. Briefly, homogeneous honey samples devoid of air bubbles were transferred into a cuvette with a 10 mm light path until the cuvette was approximately half full and the cuvette was inserted into a color photometer (HI 96785, Hanna Instruments, Cluj County, Romania). The color grades were expressed in millimeter (mm) Pfund grades when compared to an analytical-grade glycerol standard. The measurements were performed in triplicate for each sample using the approved color standards of the United States Department of Agriculture (USDA) [23].

#### 3.3.5. Color Intensity (ABS_450_)

The mean absorbance of the honey samples was determined using the method described in [5]. Briefly, the honey samples were diluted to 50% (w/v) with warm (45–50 °C) Milli-Q water and the resulting solution was filtered using a 0.45 µm filter to remove the large particles. The absorbance was measured at 450 and 720 nm using a spectrophotometer and the difference in absorbance was expressed as mAU.

### 3.4. Analysis of Antioxidant Properties

#### 3.4.1. Determination of Total Phenolic Compounds

The concentration of the phenolic compounds in the honey samples was estimated using a modified spectrophotometric Folin-Ciocalteu method [39]. Briefly, honey extract (1 mL) was mixed with Folin and Ciocalteu’s phenol reagent (1 mL). After 3 min, Na_2_CO_3_ (1 mL, 10%) solution was added to the mixture and adjusted to 10 mL with distilled water. The reaction was kept in the dark for 90 min, after which the absorbance was read at 725 nm using a T 60 UV/VIS spectrophotometer (PG Instruments Ltd, Leicestershire, UK). Gallic acid was used to calculate a standard curve (20, 40, 60, 80 and 100 μg/mL; r^2^ = 0.996). The concentration of the phenolic compounds was measured in triplicate. The results were reported as the mean ± standard deviation and are expressed as mg of gallic acid equivalents (GAEs) per kg of honey.

#### 3.4.2. Determination of Total Flavonoid Content

The total flavonoid content in each honey sample was measured using the colorimetric assay as developed by Zhishen, Mengcheng, and Jianming, [40]. Briefly, honey extract (1 mL) was mixed with distilled water (4 mL). At the baseline, NaNO_2_ (0.3 mL, 5%, w/v) was added. After 5 min, AlCl_3_ (0.3 mL, 10% w/v) was added, followed by the addition of NaOH (2 mL, 1 M) 6 min later. The volume was then increased to 10 mL by the addition of distilled water (2.4 mL). The mixture was vigorously shaken to ensure adequate mixing and the absorbance was read at 510 nm. A calibration curve was created using a standard solution of catechin (20, 40, 60, 80 and 100 μg/mL; r^2^ = 0.998). The results were expressed as mg catechin equivalents (CEQ) per kg of honey.

#### 3.4.3. DPPH Free Radical-Scavenging Activity

The antioxidant properties of each honey sample were also investigated by evaluating the free radical-scavenging activity of the DPPH radical, which was based on the method proposed by Ferreira *et al.* [2]. Briefly, honey extract (0.5 mL) was mixed with methanolic solution (2.7 mL) containing DPPH radicals (0.024 mg/mL). The mixture was vigorously shaken and left to stand for 15 min in the dark in order for the absorbance to stabilize. The reduction of the DPPH radical was determined by measuring the absorbance of the mixture at 517 nm.

Butylated hydroxytoluene (BHT) was used as a reference. The radical-scavenging activity (RSA) was calculated as the percentage of DPPH discoloration using the following equation:

% RSA = ([A_DPPH_– A_S_]/A_DPPH_) × 100 (1)

where A_S_ is the absorbance of the solution when the sample extract was added at a particular level and A_DPPH_ is the absorbance of the DPPH solution.

#### 3.4.4. Ferric Reducing/Antioxidant Power Assay (FRAP Assay)

The FRAP assay was performed according to a modified method described by Benzie and Strain [41]. Briefly, properly diluted honey (200 μL, 0.1 g/mL) was mixed with FRAP reagent (1.5 mL). Then, the reaction mixture was incubated at 37 °C for 4 min and its absorbance was read at 593 nm against a blank that was prepared with distilled water. Fresh FRAP reagent was prepared by mixing 10 volumes of 300 mM/L acetate buffer (pH 3.6) with 1 volume of 10 mM TPTZ solution in 40 mM/L HCl containing 1 volume of 20 mM ferric chloride (FeCl_3_・6H_2_O). The resulting mixture was then pre-warmed at 37 °C. A calibration curve was prepared using an aqueous solution of ferrous sulfate (FeSO_4_・7H_2_O) at 100, 200, 400, 600 and 1000 μM/L. The FRAP values were expressed as micromoles of ferrous equivalent (μM Fe [II]) per kg of honey.

#### 3.4.5. Proline Content

The proline content of the honey samples was measured using a method established by the International Honey Commission [42]. Briefly, honey (approximately 5 g) was transferred into a beaker and was dissolved in water (50 mL). The solution was quantitatively transferred to a 100 mL volumetric flask before further dilution to 100 mL with distilled water. Then the sample solution (approximately 0.5 mL) was transferred into a tube while water (0.5 mL, blank test) was transferred into a second tube; the proline standard solution (0.5 mL) was placed in three other tubes. Formic acid (about 1 mL) and ninhydrin solution (1 mL) were added to each tube. The tubes were capped carefully and shaken vigorously for 15 min. The tubes were then placed in a boiling water bath for 15 min and were immersed below the level of the solution. The tubes were further transferred to another water bath and incubated at 70 °C for 10 min. Approximately 5 mL of 2-Propanol-water solution (50% v/v) was added to each tube, followed by immediate capping. The tubes were left to cool for approximately 45 min after they were removed from the 70 °C water bath and the absorbance was measured at 510 nm (near the maximum).

### 3.5. Statistical Analyses

Assays were performed in triplicate and the results were expressed as mean values with standard deviations (SD). The significant differences represented by letters were obtained by a one-way analysis of variance (ANOVA) followed by Tukey’s honestly significant difference (HSD) post hoc test (*p* < 0.05). Correlations were established using Pearson’s correlation coefficient (r) in bivariate linear correlations (*p* < 0.01). These correlations were calculated using Microsoft office Excel 2007 and SPSS version 18.0 (SPSS Inc., Chicago, IL, USA).

## 4. Conclusions

Acacia honey samples collected in the beginning of the year (January) showed the highest color intensity and were dark amber in color; these samples contained the highest concentration of phenolics and flavonoids, as well as the highest percentage of DPPH inhibition and the highest FRAP value, confirming its high antioxidant potential. The positive correlation between DPPH and total phenolic content suggests that phenolics are the strongest contributing factor to the radical scavenging activity of the investigated Malaysian acacia honeys. Overall, our results indicated that there are seasonal variations in the antioxidant potentials of honey. Therefore, the time of honey collection is important and Malaysian acacia honey should be collected during the dry seasons, such as in January, May and June.
